# Committees Appointed at the Meeting of the American Med. Association, May, 1852

**Published:** 1852-07

**Authors:** 


*Committees appointed at the meeting of the American Medical Association, May*, 1852.—The following are the names of the members of the several committees to report at the meeting of the Association in 1853:

1. Dr. D. F. Condie, of Philadelphia—On the Causes of Tubercular Disease.
2. Dr. James Jones, of New Orleans—On the Mutual Relations of Yellow and Bilious Remittent Fever.
3. Dr. R. S. Holmes, of St. Louis, Mo.—On Epidemic Erysipelas.
4. Dr. Charles D. Meigs, of Philadelphia—On Acute and Chronic Diseases of the Neck of the Uterus.
5. Dr. J. P. Jervey, of Charleston, S. C.—On Dengue.
6. Dr. Daniel Drake, of Cincinnati, O.—On Milk Sickness, so called.
7. Dr. A. Lopez, of Mobile, Ala.—On the prevalence of Idiopathic Tetanus.
8. Dr. Geo. B. Wood, of Philadelphia—On Diseases of the Parasitic Organs.
9. Dr. R. D. Arnold, of Savannah, Ga.—On the Physiological Peculiarities and Diseases of Negroes.
10. Dr. Joseph Carson, of Philadelphia—On the Alkaloids which may be substituted for Quinia.
11. Dr. S. D. Gross, of Louisville, Ky.—On Results of Surgical Operations for the Relief of Malignant Diseases.
12. Dr. James R. Wood, of New York—On Statistics of the Operation for the removal of Stone in the Bladder.
13. Dr. Alexander H. Stevens, of New York—On Sanitary Principles applicable to the Construction of Dwellings.
14. Dr. F. Peyre Porcher, of Charleston, S. C.—On Toxicological and Medicinal Properties of our Cryptogamic Plants.
15. Dr. G. Emerson, of Philadelphia—On Agency of the Refrigeration produced through Upward Radiation of Heat as an exciting cause of Disease.
16. Dr. Henry J. Bigelow, of Boston, Mass.—On the Best Means of Making Pressure in Reducible Hernia.
17. Dr. A. T. B. Merritt, of Richmond, Va.—On Cholera and its Relation to Congestive Fever—their analogy or identity.
18. Dr. Usher Parsons, of Providence, R. I.—On Displacements of the Uterus.
19. Dr. H. F. Campbell of Augusta, Ga.—On Typhoid Fever.
20. Dr. Worthington Hooker, of Conn.—On Epidemics of New England and New York.
21. Dr. John L. Atlee, of Lancaster, Pa.—On Epidemics of New Jersey, Pennsylvania, Delaware and Maryland.
22. Dr. Robert W. Haxall, of Richmond, Va.—On Epidemics of Virginia and North Carolina.
23. Dr. Wm. M. Bolling, of Montgomery, Ala.—On Epidemics of South Carolina, Georgia, Florida and Alabama.
24. Dr. Edward H. Barton, of New Orleans, La.—On Epidemics of Mississippi, Louisiana, Texas and Arkansas.
25. Dr. W. L. Sutton, of Georgetown, Ky.—On Epidemics of Tennessee and Kentucky.

26. Dr. Thomas Reyburn, of St. Louis, Mo.—On Epidemics of Missouri, Illinois, Iowa and Wisconsin.

27. Dr. George Mendenhall, of Cincinnati, Ohio—On Epidemics of Ohio, Indiana and Michigan.

*Committee on Volunteer Communications*—Drs. Joseph M. Smith, John A. Swett, Willard Parker, Gurdon Buck and Alfred C. Post, of New York

